# Spontaneous renal subcapsular fluid accumulation with hemorrhage due to ureteral stone: a case report and literature review

**DOI:** 10.3389/fsurg.2025.1580745

**Published:** 2025-05-21

**Authors:** Yudong Hu, Xiaofeng Wang, Yujie Chen, Jun Li, Faming Zhu, Ye Yuan, Jin Ye, Fan Yang, Yong Zhong

**Affiliations:** Department of Urology, The Thirteenth People’s Hospital, Chongqing, China

**Keywords:** spontaneous, renal subcapsular fluid, renal subcapsular hemorrhage, ureteral stone, case report

## Abstract

**Background:**

Subcapsular or perirenal hemorrhage is a serious complication commonly associated with exogenous trauma and medical interventions. However, spontaneous subcapsular or perirenal hemorrhage can occur in the absence of known trauma, presenting as a rare but potentially life-threatening urological condition.

**Case presentation:**

This case report describes a 59-year-old female patient who presented with left flank pain as the main symptom, with no history of trauma. An enhanced abdominal computerized tomography (CT) scan revealed a left upper ureteral stone, severe left hydronephrosis, and left renal subcapsular fluid accumulation with hemorrhage. Following 2 weeks of conservative treatment, the patient underwent double-J ureteral stent insertion after stabilization of the left renal subcapsular hemorrhage. The stent was regularly replaced, and follow-up CT scans were conducted. After the resolution of left renal pelvic effusion and absorption of the left renal subcapsular fluid with hemorrhage, the patient underwent retrograde intrarenal surgery (RIRS), leading to successful treatment.

**Conclusions:**

In cases of spontaneous renal subcapsular fluid accumulation with hemorrhage due to ureteral stone, conservative treatment through ureteral stent placement for renal preservation is worthwhile. Then, management of ureteral stone by second-stage RIRS after absorption of the renal subcapsular hemorrhage is an available option.

## Introduction

Renal subcapsular or perirenal fluid accumulation is a common finding in patients with renal colic and urinary stones, particularly in cases involving hydronephrosis and ureteral stone obstruction (1). Renal subcapsular hemorrhage (RSH) or perirenal hemorrhage (PRH) is usually associated with exogenous trauma and can also occur as a complication of medical interventions, such as urologic surgery (2, 3). Spontaneous renal subcapsular hemorrhage (SRSH) or spontaneous perirenal hemorrhage (SPRH) refers to bleeding into the subcapsular or perirenal space as a result of the rupture of renal parenchyma or renal peripheral blood vessels, occurring without a history of trauma (4). SRSH and SPRH occur predominantly in patients with renal tumors, renal vascular lesions, hydronephrosis, renal infections, and those undergoing hemodialysis (5). SRSH and SPRH, though rare, are potentially life-threatening urological disorders, usually characterized by acute flank pain, a palpable mass, and hypotensive shock (6). PRH has also been reported as a rare complication of renal ureteral stones (7). However, cases of SRSH specifically caused by ureteral stones remain limited. In this study, we report a case of spontaneous renal subcapsular fluid accumulation with hemorrhage due to ureteral stone obstruction, which was successfully cured.

## Case presentation

**Figure 1:**
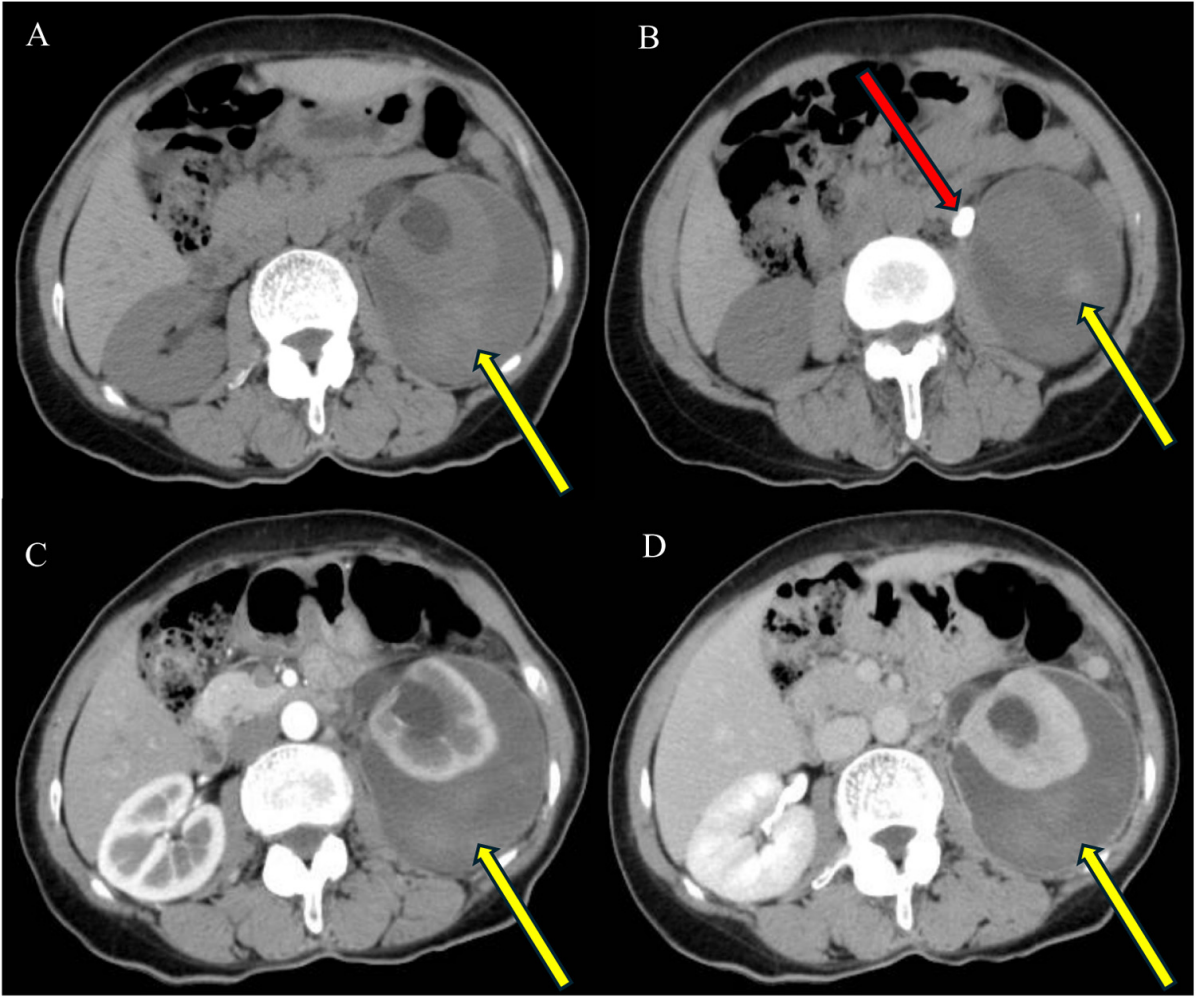
On plain CT images **(A,B)**, the left renal pelvis appears dilated, with fluid encapsulation around the left kidney containing flaky hyperdense shadows (yellow arrows), and one stone is visible in the left upper ureter (red arrow). On enhanced CT images **(C,D)**, no enhancing lesions are observed, and no contrast leakage is detected in the excretory phase scan **(D)**.

**Figure 2:**
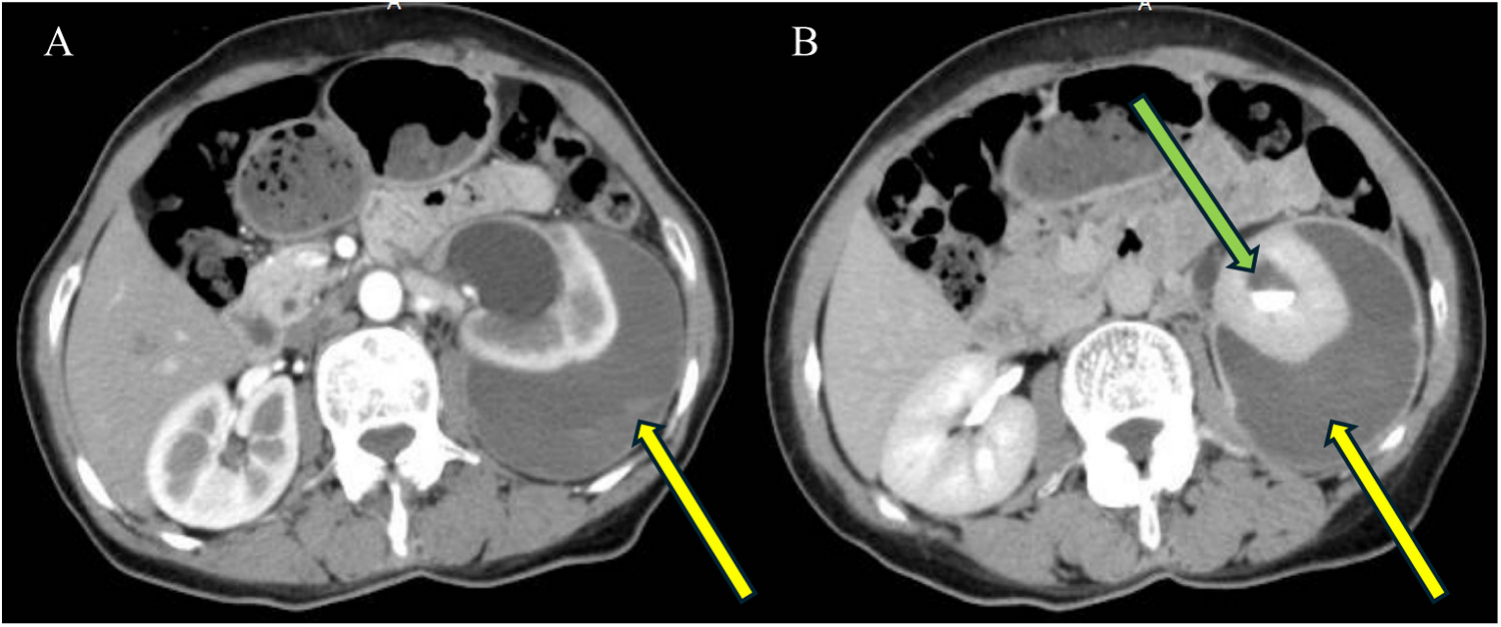
Enhanced CT **(A,B)** after 2 weeks of conservative treatment shows dilatation of the left renal pelvis, fluid encapsulation around the left kidney, a flocculent hyperdense shadow (yellow arrow) under the encapsulation, no enhancement, and compression of adjacent organs. The excretory phase scan reveals contrast in the left renal pelvis (**B**, green arrow) with no contrast leakage.

**Figure 3:**
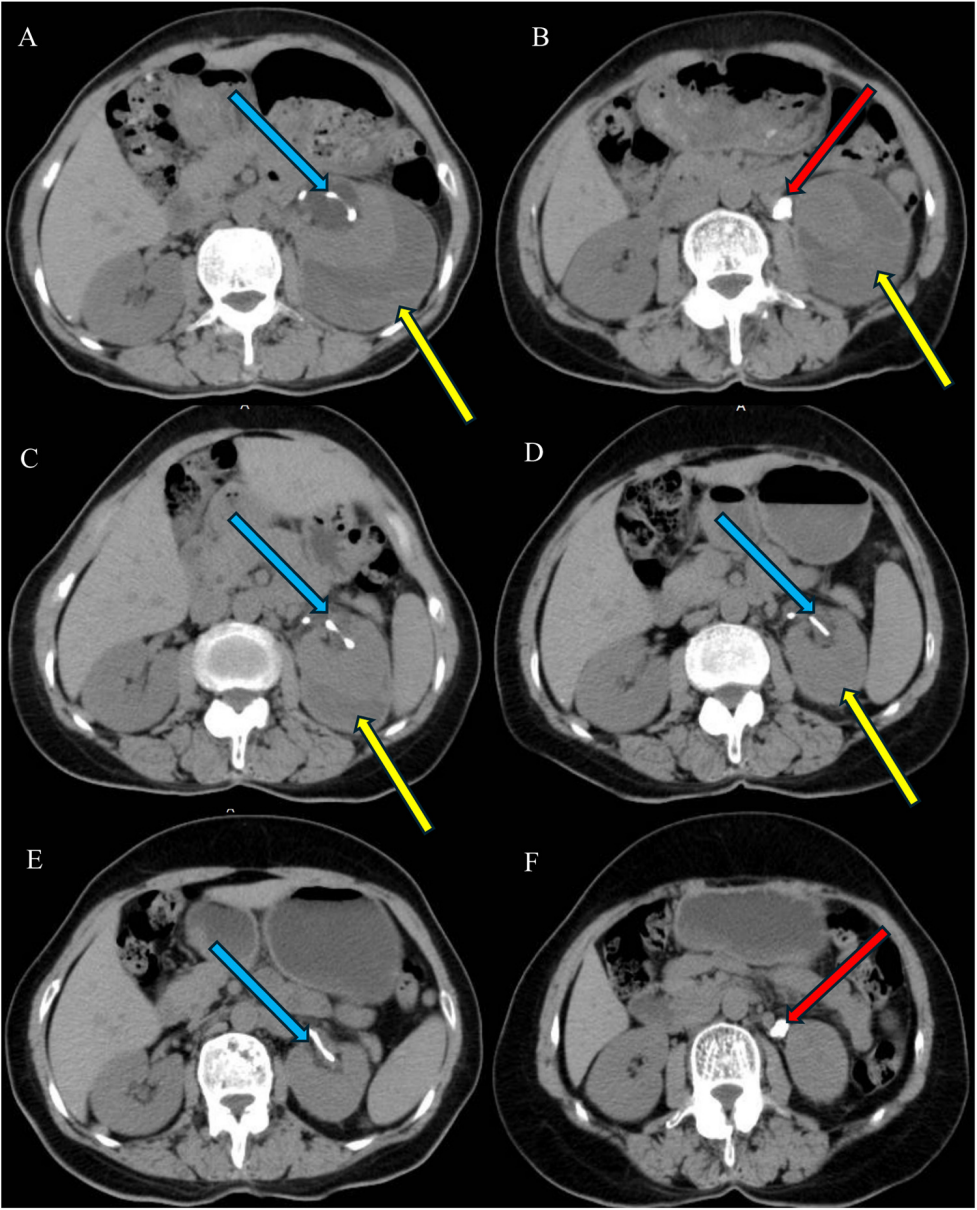
One week after ureteral stent insertion **(A,B)**: A double-J ureteral stent is observed in position in the left renal pelvis (blue arrows), with fluid encapsulation and flocculent hyperdense shadows surrounding the left kidney **(A,B)**. CT on day 47 shows a decrease in the left renal subcapsular fluid, with the hematoma largely absorbed **(C)** Repeat CT on day 82 reveals mild dilatation of the left renal pelvis, with the left renal subcapsular fluid mostly absorbed **(D)** CT on day 127 shows no abnormalities in the left renal parenchyma, disappearance of fluid in the left renal pelvis, and complete absorption of the left renal subcapsular hematoma **(E,F)**.

A 59-year-old woman presented with left flank pain persisting for 20 days, with no history of trauma, fever, or hematuria. This patient had occasional elevated blood pressure in the past, but had not gone to the hospital for a definitive diagnosis of hypertension and had not taken antihypertensive medications or regular blood pressure monitoring. The patient had no history of acute or chronic renal disease, no history of diabetes mellitus, and had not taken any antiplatelet or anticoagulant medications. The patient had no previous history of urinary stones and had not undergone any abdominal or urologic surgery. At the time of admission, the patient's vital signs were stable (blood pressure 162/105 mmHg, heart rate 87 beats per min, and body temperature 36.5°C). The hemoglobin level was 122 g/L (normal range: 115–150 g/L), hematocrit was 40.00% (normal range: 35%–45%), blood urea nitrogen was 6.27 mmol/L (normal range: 3.10–8.80 mmol/L), and creatinine level was 81.0 μmol/L (normal range: 45–125 μmol/L). The prothrombin time was 13.7 s (normal range: 9.0–14 s), international normalized ratio was 0.97 (normal range: 0.80–1.50), activated partial thromboplastin time was 30.60 s (normal range: 23.30–32.50 s), and thrombin time was 18.5 s (normal range: 14–21 s). Routine urine tests were generally normal, with no significant urinary red or white blood cells. An enhanced abdominal CT scan revealed a 2.0 cm × 1.3 cm stone in the left upper ureter, severe left renal pelvic hydronephrosis, and fluid accumulation around the kidney. Additionally, a flocculent, slightly high-density shadow was observed under the left renal capsule without enhancement, compressing adjacent organs. The patient was diagnosed with (1) left upper ureteral stone with left renal pelvic hydronephrosis and (2) left renal subcapsular fluid accumulation with hemorrhage (see Figure 1). The patient was treated conservatively, and on the day after admission, her retested hemoglobin level did not decrease, the renal function tests were normal, there was no gross hematuria, and the vital signs were stable. For the next two weeks, the patient remained on absolute bed rest while vital signs were monitored. Symptom progression was observed, and measures were taken to prevent infections. Routine blood tests, urinalysis, renal function assessments, and other relevant indicators were intermittently rechecked. On day 14, a repeat CT scan showed that the left renal subcapsular hemorrhage had decreased insignificantly in size but had not progressed (see Figure 2). The retested hemoglobin level was 127 g/L, hematocrit was 40.50%, blood urea nitrogen was 7.51 mmol/L, and creatinine level was 81.0 μmol/L. Vital signs monitoring showed continued stability. Her left flank pain lessened over time, and also without gross hematuria. Given the hemorrhage's stability, the patient underwent the first left double-J ureteral stent insertion. A follow-up CT on day 23 (one week after stent insertion) showed slight resorption and reduction in the left renal subcapsular fluid accumulation with hemorrhage (Figures 3A,B), and the patient was discharged. By day 47, the left renal subcapsular fluid had further decreased, and the subcapsular hematoma was largely absorbed (Figure 3C). At this stage, the left double-J ureteral stent was replaced. On day 82, CT imaging showed mild left renal pelvic dilation and significant absorption of the left renal subcapsular fluid (Figure 3D), leading to a second replacement of the left double-J ureteral stent. A follow-up CT on day 127 (Figures 3E,F) revealed no abnormalities in the left renal parenchyma, no dilation of the left renal pelvis, and complete resolution of renal pelvic effusion. After preoperative preparation, the patient underwent retrograde intrarenal surgery (RIRS) on day 138. At follow-up, the patient reported no significant discomfort. The treatment was successful, and the patient achieved full recovery.

## Discussion

SRSH and SPRH occurring in the absence of known trauma are collectively referred to as Wunderlich syndrome (WS) (8). The primary causes of SRSH and SPRH include renal tumors, renal cysts, renal arteriovenous malformations, renal aneurysms, hydronephrosis, and chronic renal disease, with renal tumors being the most common etiology (9, 10). Literature reports indicate that ureteral or renal stone obstruction can lead to spontaneous renal rupture, which may result in urinary extravasation, SRSH, and SPRH (11, 12). In this case, the patient presented with upper ureteral stone obstruction and severe hydronephrosis, with no history of trauma, anticoagulant use, or significant past medical conditions apart from hypertensive disorders. Enhanced CT imaging did not reveal any renal masses, renal vascular lesions, or abdominal or pelvic tumors. Therefore, we consider this case a spontaneous renal subcapsular fluid accumulation with hemorrhage caused by ureteral stone obstruction.

There are three possible pathophysiologic mechanisms. 1. Upper urinary tract obstruction due to urinary stones can lead to increased intrarenal pressure and renal parenchymal fragility, which increases the risk of spontaneous parenchymal rupture (13, 14). The most common cause of renal forniceal rupture is obstructive ureteral lithiasis (15). Acute ureteral occlusion by a stone leads to a sharp increase in collecting system pressure, exceeding the compensatory mechanisms of the renal pelvis and ureter. This pressure overload causes rupture at the weakest anatomical site—the renal fornix (16). Rupture of renal fornix leads to extravasation of urine from the calyx into the subcapsular, perirenal, or retroperitoneal space and may cause rupture of small blood vessels in the renal parenchyma, resulting in hemorrhagic extravasation and hematoma formation. When urine extravasation follows, the release of pressure leads to pain relief and a decrease in the number of days with significant symptoms, sometimes described as a protective renal self-help mechanism (17–19). 2. Hydronephrosis leads to a sudden change in pressure within the renal pelvis, increasing renal venous pressure and dilation of the veins around the kidneys, causing rupture of small blood vessels and inducing RSH and PRH (20). 3. Infections and impaired renal function due to obstruction can induce pathological changes in the kidney, increasing its susceptibility to rupture, even in the absence of significant trauma (21).

Acute onset of flank pain, lumbar abdominal mass, and hypovolemic shock (Lenk's triad) are typical manifestations of WS but are seen in less than a quarter of patients, with the majority of patients presenting with flank pain only and even asymptomatic subcapsular hematomas (8). The exact presentation depends on the size of the hematoma, the compression exerted on the surrounding parenchyma, and the degree of blood loss (22). It can also become infected and form a perinephric abscess or sepsis, and both bleeding and infection can be life-threatening (23). In a study analysis, 92% of patients presented with flank pain, 8% with visible hematuria, 39% with microscopic hematuria, and 23% exhibited unstable vital signs due to shock (24). Cullen and Grey Turner signs have also been reported in patients with idiopathic perirenal hemorrhage (25). The pressure exerted by the hematoma on the peripheral parenchyma can induce renal hypoperfusion and ischemia, leading to activation of the renin–angiotensin–aldosterone axis, which can induce refractory hypertension (26). In patients with preexisting renal impairment, such as those with chronic kidney disease or a solitary kidney, RSH may further impair renal function, sometimes leading to acute renal failure (22).

Ultrasound, CT, and MRI are the main tests for SRSH and SPRH (27). Ultrasound is the simplest and quickest diagnostic modality, with the advantage of real-time bedside monitoring of hematoma changes, but it is usually less accurate (28). CT scanning remains the preferred test for the diagnosis of SRSH and SPRH and is capable of characterizing the potential etiology (10, 29). However, in up to 60% of cases, initial CT may miss renal cancer, and a post-treatment review of CT imaging can help confirm the diagnosis and assess for persistent bleeding and urinary extravasation (30). MRI is an alternative to CT, and it has been suggested that a combination of CT and MRI can be used to accurately diagnose SPRH (31). Arterial angiography can be used to diagnose or treat some patients, to assess for active arterial extravasation, and to help identify vascular abnormalities and when embolization is required (32).

For SRSH and SPRH, the choice of emergency surgical treatment, such as ureteral stent insertion, percutaneous drainage, hematoma debridement surgery, nephrectomy, interventional embolization, or conservative treatment, remains controversial. We reviewed the literature on this group of diseases and identified 41 cases (7, 11–14, 19, 21, 33–35), and a brief summary of these cases is listed in the Table 1.

**Table 1:** Characteristics of SRSH and SPRH cases associated with urinary stones.

First author	Age(y)	Sex	Cases	Time	Infection	LBP	Anemia	Management	Outcome
Chaudhary (21)	35	M	1	NA	Yes	Yes	Yes	EN	CR
Chaabouni (14)	61	F	1	2 weeks	No	No	NA	EURL	CR
Petros (7)	70	M	1	24 h	Yes	Yes	Yes	EN	CR
Ufuk (12)	41	M	1	5 days	Yes	No	No	EUSI; PCED	CR
Setia (33)	57 ± 13.1	12M/20F	32	1.61 days	10/32	NA	NA	CM 16, EURS 6, EUSI 6, PCN 4	CR
Chiancone (13)	64	M	1	4 weeks	Yes	Yes	Yes	EN	Dead
Prem (11)	30	F	1	1 month	Yes	Yes	Yes	EN	CR
Yin (34)	67	M	1	NA	Yes	No	Yes	RN	CR
Suarez (19)	67	M	1	6 h	No	No	NA	CM	CR
Toyoshima (35)	54	M	1	NA	No	No	No	EUSI, CM, URL (6 months)	CR
This case	59	F	1	20 days	No	No	No	DUSI, CM, RIRS (138 days)	CR

M, Male; F, Female; Time, Time to diagnosis after trigger event; LBP, Low blood pressure; EN, Emergency nephrectomy; URL, Ureteroscopic lithotripsy; EURL, Emergency ureteroscopic lithotripsy; DUSI, Delayed ureteral stent insertion; EUSI, Emergency ureteral stent insertion; PCED, Percutaneous external drain; CM, Conservative management; EURS, Emergency ureteroscopy; PCN, Percutaneous nephrostomy; RN, Radical nephrectomy; CR, Complete resolution; RIRS, Retrograde intrarenal surgery; NA, Not available.

The management of SRSH and SPRH depends on the underlying etiology, hemodynamic status, and severity, as both conditions can lead to life-threatening hemorrhage and hypovolemic shock. Conservative, nonsurgical management is typically considered in patients with stable hemodynamics, stable vital signs, no evidence of sustained blood loss, no progression of anemia, absence of severe complications, and no renal tumors (19, 33, 36–38). Immediate or delayed ureteral stent insertion is the most common urologic intervention in addition to conservative treatments such as bed rest, rehydration, blood transfusion, and infection prevention (33, 35, 38). In cases where patients do not respond to resuscitation and transfusion, prompt surgical intervention is required to prevent mortality (7). Nephrectomy may be considered for rapidly progressing disease, severe hemorrhage, or cases where CT imaging suggests renal tumor rupture with bleeding (11, 13, 21, 24, 34). Additional treatment options include emergency percutaneous drainage, renal capsular hematoma dissection (39), and selective angiographic embolization (40, 41). Reports have also described successful management with percutaneous drainage of the hematoma, percutaneous nephrolithotomy with ultrasonic negative pressure hematoma aspiration, and combined urokinase injection following cessation of bleeding (42, 43).

In this case, early surgical intervention was not considered due to the absence of active bleeding, stable vital signs, lack of anemia or shock, and no evidence of a renal tumor. The patient showed clinical improvement following double-J ureteral stent insertion. Immediate or delayed ureteral stent insertion is the most commonly performed procedure for managing injuries to the collecting system; however, the optimal timing for decompression remains unclear (38). An emergency decompression procedure, such as immediate ureteroscopic lithotripsy or immediate ureteral stent insertion, was not performed due to uncertainty regarding the acuity and stability of the bleeding. When RSH occurs, the renal parenchyma is usually compressed by hemorrhagic fluid accumulating in the subcapsular space, which may provide a temporary hemostatic effect. If ureteroscopic lithotripsy is performed immediately, there is a risk of rupture and hemorrhage from sudden decompression of the compressed and deformed blood vessels around the kidney and within the renal parenchyma (44, 45). This mechanism is similar to hematuria caused by catheterization for acute urinary retention, in which the submucosal veins of the bladder wall are suddenly and rapidly constricted by the dilated bladder, leading to bleeding and hematuria (46). Additionally, hematuria and renal subcapsular hematoma have been reported in patients with urinary retention due to an indwelling catheter (47). Therefore, the patient initially underwent 2 weeks of bed rest and conservative treatment. Repeat CT imaging showed no increase in renal subcapsular fluid or hemorrhage, confirming stability, after which the first ureteral stent was inserted. Subsequent follow-up CT scans demonstrated gradual resolution of left hydronephrosis, left renal subcapsular fluid, and hemorrhage. The ureteral stent was replaced at regular intervals, and the final follow-up CT showed that the left hydronephrosis disappeared and that the left renal subcapsular fluid and hemorrhage were completely absorbed and dissipated. The patient subsequently underwent RIRS and was discharged without recurrence.

## Conclusion

In cases of spontaneous renal subcapsular fluid accumulation with hemorrhage due to ureteral stone, conservative treatment through double-J ureteral stent placement for renal preservation is worthwhile. Then, management of ureteral stone by a second-stage RIRS after CT evaluation of absorption of renal subcapsular hemorrhage is an available option.

## Data Availability

The original contributions presented in the study are included in the article/Supplementary Material, further inquiries can be directed to the corresponding author.
